# Prevalence of Dental Trauma in Spain: Systematic Review and Meta‐Analysis

**DOI:** 10.1002/cre2.70128

**Published:** 2025-04-02

**Authors:** Elda Esther García Méndez, David Ribas‐Pérez, Diego Rodríguez Menacho, Ignacio Barbero Navarro, Eva Rosel Gallardo, Antonio Castaño Séiquer

**Affiliations:** ^1^ Facultad de Odontología Universidad de Sevilla Seville Spain; ^2^ Facultad de Odontología Universidad de Granada Granada Spain

**Keywords:** adolescents, children, dental fractures, meta‐analysis, prevalence, Spain, systematic review, traumatic dental injuries

## Abstract

**Objectives:**

To estimate the prevalence of TDI in the Spanish population through a systematic review and meta‐analysis, identifying differences by sex, age, and study setting.

**Materials and Methods:**

An exhaustive search was conducted in databases such as PubMed, Scopus, Embase, Ovid Medline, and CINAHL, including gray literature and other alternative sources. Observational studies evaluating the prevalence of TDI in Spain, with a total of 8662 participants. The methodological quality of the studies was assessed using the JBI tool, and the PRISMA guidelines were followed to ensure transparency and reproducibility.

**Results:**

The estimated overall prevalence of TDI was 9.94% (95% CI: 5.98%–16.6%). The results showed a higher prevalence in males (10.5%) compared to females (5.7%), and in children (11.1%) compared to adolescents (6.1%). Fractures were the most common type of TDI (56.5%), followed by avulsion (4.0%). High heterogeneity was observed among the studies, suggesting variability in data collection methods and TDI classification.

**Conclusions:**

This study is the first to estimate the prevalence of TDI in Spain, which sheds light on the need for a standardized approach in future research. Although it presents significant methodological strengths, limitations such as high heterogeneity and lack of standardization should be considered when interpreting the results.

## 1 Introduction

Traumatic dental injuries (TDI) are the second most common oral disease after dental caries, affecting between 900 million and 1.2 billion people worldwide, of which approximately 180 million are children under 6 years of age (Petti, Glendor, et al. 2018; Petti, Andreasen, et al. 2018).

According to a meta‐analysis by Petti, Glendor, et al. (2018), it ranks fifth in the list of the most prevalent diseases or traumatic injuries with 15.2% globally, after dental caries, tension headache, iron deficiency anemia and hearing loss.

The prevalence of TDI varies considerably between regions, being 14% in the European region (Petti, Glendor, et al. 2018); but it also varies between countries and even within each country as a consequence not only of the socioeconomic and cultural diversity or the customs and lifestyles of each society, but also due to the absence of a standardized system for its registration and classification due to the variety of classification systems: Andreasen, O'Brien, Ellis; and added to the fact that many researchers create their own classification systems or modify existing ones (Feliciano and Caldas 2006). These limitations are expected to diminish following the approval by the World Health Organization (WHO) in 2022 of the NAOD classification for TDI (injury to teeth or supporting structures) based on the Andreasen classification (Petti et al. 2022).

TDI has a high incidence in pre‐school children between 2 and 3 years of age with no gender differences due to the development of motor coordination at this stage (Oldin et al. 2015), and between 8 and 10 years of age with a predominance of males due to their propensity for risky and more aggressive behavior, high involvement in physical activities and active sports (Bratteberg et al. 2018; Lembacher et al. 2022).

The anterosuperior teeth are the most affected by TDI, both in the primary and permanent dentition, predominantly in the group of upper incisors, due to their anatomical position protruding in front of the lower incisors and the direction of the impacts that generally occur from the front (Lam 2016). On the other hand, the presence of malocclusions and deforming oral habits (digital sucking and mouth breathing) are associated with a significantly higher frequency of traumatic dental injuries (Al‐Batayneh et al. 2017; Jeyashree et al. 2022). Studies by Gupta et al. (2011) and Basha et al. (2021) conclude that children with increased overjet (> 3 mm) are 4–5 times more likely to develop TDI, while those with inadequate lip coverage are 2–3 times more at risk.

Dental trauma has a negative impact on the quality of life in relation to oral health, whether in children, adolescents or adults. The repercussions not only include the physical‐inflammatory alterations inherent to the injury, such as pain and functional impotence, but also esthetic alterations, highly costly treatment for the individual and for the national system, and a strong psychological burden with a negative impact on quality of life (Milani et al. 2021; El‐Kalla et al. 2017). A very important aspect is the repercussions on the development of successive permanent teeth, with possible sequelae such as coronary, root and enamel defects, root dilacerations, pulp obliterations, necrosis and delays in tooth development and eruption (Siahi‐Benlarbi et al. 2012).

According to data provided by the WHO in its reports on the state of oral health (WHO 2023), in Europe the annual expenditure due to oral diseases amounts to about 113 billion dollars, the second highest figure among all regions, but within these oral conditions the WHO does not include TDI despite its high prevalence in the world.

With this we can affirm that it is a neglected condition and to a certain extent undervalued by those responsible for health policies, making it difficult to allocate adequate resources and implement preventive programmes. It is therefore necessary for each country to take a detailed look at the trend, presentation patterns and risk factors of TDI, to establish comparisons with other countries, formulate health policies and improve emergency care, reducing costs in the long term, which leads us to ask the following question: What is the prevalence of dentoalveolar trauma in Spain?

With the aim to estimate the prevalence of TDI in the Spanish population through a systematic review and meta‐analysis this study was made.

## 2 Materials and Methods

The guidelines of the Preferred Reporting Items for Systematic Reviews and Meta‐Analyses (PRISMA) (Page et al. 2021) were followed for the preparation of the final research report. The protocol was published on the International Platform for Registered Protocols for Systematic Reviews and Meta‐Analyses (INPLASY), registration number INPLASY202480076 (García Méndez et al. 2024).

### 2.1 Selection Criteria

We included observational studies that examined the prevalence of dentoalveolar trauma in the Spanish population. The studies had to meet the following criteria: cross‐sectional or cohort design; Spanish participants or long‐term residents in Spain; assessment of the prevalence of dental trauma; publication in Spanish, English, Basque, Catalan, Galician, or Valencian; and definition of the criteria used to define the types of trauma. The clinical context in which the study was conducted was also considered, since research carried out in emergency departments may tend to overestimate the prevalence of TDI. Those that analyzed trauma in the context of craniofacial anomalies, neurological disorders or systemic diseases were excluded, as well as those that focused exclusively on sports‐related trauma. Case‐control studies were also excluded.

### 2.2 PEO Question

As this is a systematic review of prevalence studies, no direct comparison is specified, so the question is a PEO question, not a PECO one. The elements of the question were:

- *Population (P):* Children and adolescents participating in Spanish studies on dental trauma.
- *Exposition (E):* Traumatic dental injuries (TDI).
- *Outcome (O):* Prevalence of traumatic dental injuries (TDI).

And finally the question was:

*What is the prevalence of traumatic dental injury (TDI) in the Spanish population of children and adolescents?*

### 2.3 Sources of Information

An exhaustive search was carried out in the PubMed, Scopus, Embase, Ovid Medline and CINAHL databases. Search terms included keywords related to “dentoalveolar trauma,” “dental fracture,” “dental avulsion,” “dental luxation,” “Spain,” and among others that were identified through preliminary searches. In addition, records of preprints, reference lists of the selected articles were consulted and a search of gray literature was carried out in Google Scholar and in the repositories of bachelor's, master's and doctoral theses of Spanish universities.

### 2.4 Search Strategy

Search strategies were adapted to the characteristics of each database. These were constructed by combining controlled terms and free text terms, using Boolean operators, truncated words and wildcards. Results were limited to studies published in one of the languages already defined, without restriction by publication date. No filters were applied to the search related to study design, to identify all observational studies that met the inclusion criteria. The search strategy can be found in the published protocol (García Méndez et al. 2024).

### 2.5 Registration of Studies

#### 2.5.1 Data Management

The search results were exported to the Rayyan software (Ouzzani et al. 2016), an online tool that facilitates the selection and management of studies for systematic reviews, as well as the coordinated work of the research team. This software was used to detect and eliminate duplicate articles, as well as an initial screening through a semi‐automated data mining tool that categorized and labeled studies according to inclusion and exclusion criteria.

### 2.6 Selection Process

The selection of studies was done in two phases. In the first phase, two independent reviewers, blinded to each other's decision, assessed the titles and abstracts of the identified studies. Studies that passed this first phase were retrieved in full text and assessed in the second phase by the same reviewers, applying the inclusion and exclusion criteria. Disagreements between reviewers were resolved by consensus or, if necessary, with the involvement of a third reviewer. The sequence of the selection process was documented following the PRISMA flow chart.

#### 2.6.1 Data Extraction

A reviewer, using a standardized form, extracted the following data from the selected studies: author, year of publication, study design, sample size, age, and sex of participants, prevalence (and their 95% confidence intervals [CIs]) of dentoalveolar trauma in general and of each specific type (fractures, dislocations, and avulsions), and definition of the criteria used to classify the types of trauma. In case of incomplete or unclear data, we attempted to contact the authors of the original studies for additional information.

### 2.7 Definition of the Outcome Variable

The main variable was the prevalence of dentoalveolar trauma in the Spanish population, expressed as a percentage. The outcomes assessed were adapted from those that already reported in another meta‐analysis [1]: TDI in children, adolescents and adults, TDI for both biological sexes, TDI in the primary dentition and TDI in the permanent dentition. The setting in which the study was conducted was also considered.

### 2.8 Assessment of Methodological Quality

To assess the methodological quality of the included studies, the JBI Critical Appraisal Checklist for Prevalence Studies (Munn 2020) was used, which assesses aspects such as representativeness of the sample, sample size, data collection methods and statistical analysis. This tool has nine assessment items, each of which can be assessed as: Yes, No, Unclear and Not applicable. Two reviewers independently assessed the quality of each study, and any disagreements were resolved by consensus or with the involvement of a third reviewer.

### 2.9 Data Synthesis

From the extracted data, a random effects meta‐analysis was performed using R software (R Core Team 2021) and the “meta” package (Schwarzer et al. 2015). Overall and subgroup prevalence was calculated, with corresponding 95% confidence intervals. Studies with high heterogeneity were analyzed for possible sources of heterogeneity, and sensitivity analyses were performed to assess the impact of individual studies on the overall results. To assess heterogeneity between studies, statistical tests such as calculation of the *I* ^2^ statistic, τ2 and the prediction interval were used.

An assessment of the certainty of evidence at the outcome level was not performed because the outcome variable does not require a rating of the quality of evidence, as it is a point prevalence and not an effect. Publication bias was also not assessed due to the lack of statistical power of the available evidence when few studies are included in the meta‐analysis (Furuya‐Kanamori et al. 2018).

## 3 Results

### 3.1 Selection of Studies

**Figure 1:**
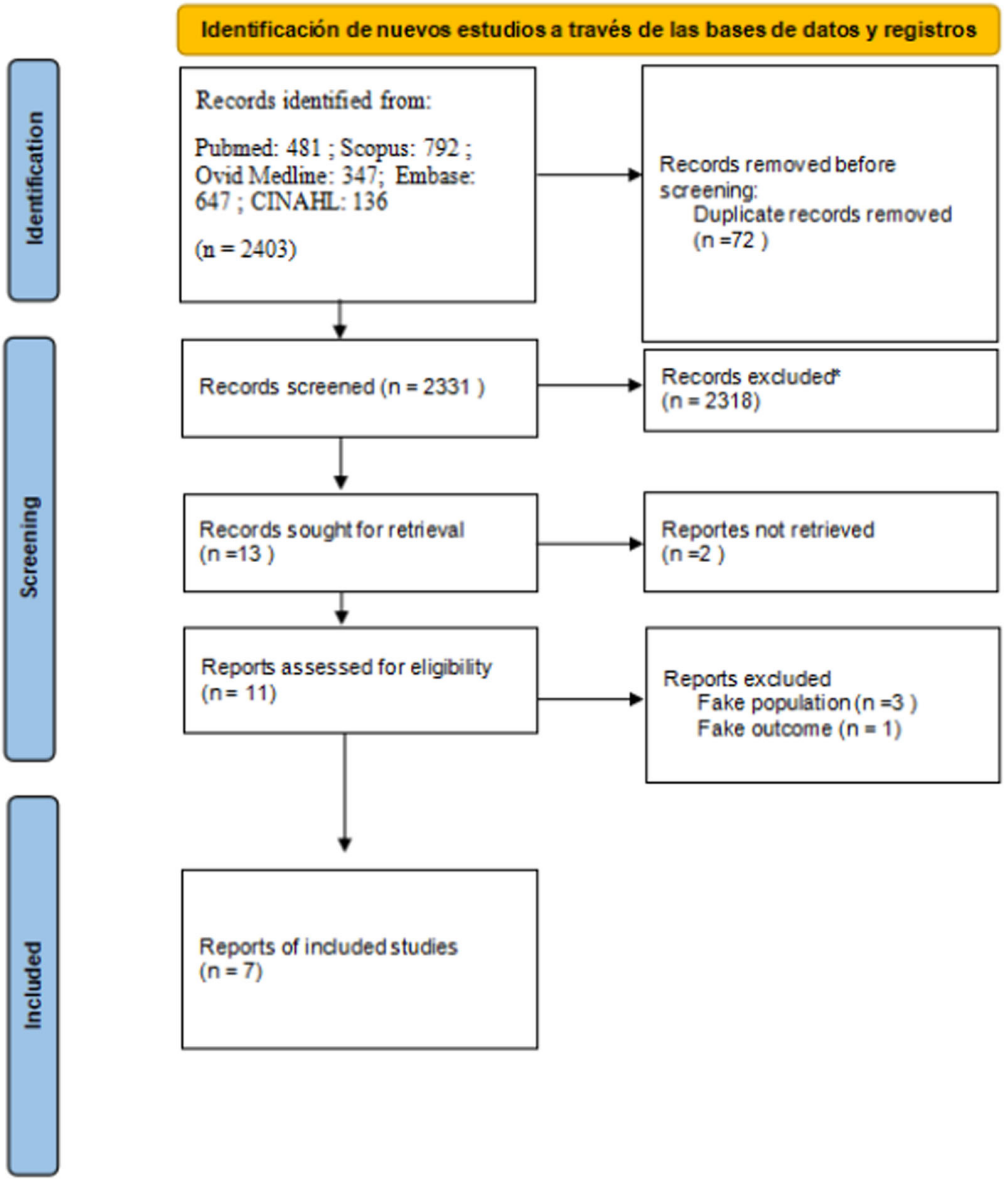
Flowchart of the study selection process.

A total of 2403 studies were identified through the five database chosen (PubMed 481, Scopus 792, Embase 647, Ovid Medline 347, and CINAHL 136). After removing duplicates, 2331 titles and abstracts were reviewed, of which 2318 were excluded as they did not meet the inclusion criteria. Of the 13 studies selected for full‐text review, only 11 could be retrieved because two of them were published in journals that were discontinued many years ago. Finally, seven studies were included in the systematic review and meta‐analysis (Zaragoza et al. 1998; Tapias et al. 2003; Segura and Poyato 2003; Faus‐Damia et al. 2011; Mendoza‐Mendoza et al. 2015; Faus‐Matoses et al. 2020; Veloso Duran et al. 2022) (Figure 1).

### 3.2 Characteristics of the Included Studies

The studies included in this review were conducted in only four Spanish provinces and involved a total population of 8662 participants. Sample sizes ranged from 251 to 4000 subjects, with a generally balanced gender distribution. The age of participants ranged from 1‐year‐old children to 77‐year‐old adults. Most studies were conducted in dental clinics. Various classification systems were used to assess dentoalveolar trauma, with the Hargreaves and Craig and Andreasen systems being the predominant ones. The types of TDI analyzed included avulsions, fractures, dislocations and intrusions, with reported prevalence ranging from 4.5% to 21.72%, reflecting heterogeneity in the methodologies and populations studied (Table 1).

**Table 1:** Caracteristics of the studies included.

Study	Study design	Location	Scope of the study	Sample size	Age of the participants	Gender	Classification used	Type of dentition	TDI	General TDI prevalence
Zaragoza et al. (1998)	Cross sectional, observational study.	Valencia	School	4000	Range: 6–12	Male: 2140 (53.5%) Female: 1860 (46.5%)	Hargreaves & Craig	Mixed	Avulsion Fracture	227 (5.7%)
Tapias et al. (2003)	Cross sectional, observational study.	Madrid	Clínic	470	10 yearss	Males: 246 (52.3%) Females: 224 (47.7%)	Only fractures	Mixed	Fracture	82 (17.44%)
Segura and Poyato (2003)	Cross sectional, observational study.	Sevilla	Kindergarden	337	3 years	Males: 192 (56.97%) Females: 145 (43.03%)	Hargreaves & Craig	Deciduous	Fracture	49 (14.54%)
Faus‐Damia et al. (2011)	Cross sectional, observational study.	Valencia	School	1325	Range: 6–18	Males: 678 (51.2%) Females: 647 (48.8%)	IADT	Permanent Mixed	Avulsion Luxation Fracture	82 (6.2%)
Mendoza‐Mendoza et al. (2015)	Cross sectional, observational study.	Sevilla	Clínic	879	Range: 1–7 años	Not specificed	Not specificed	Deciduous	Avulsion Luxation Fracture Intrusion	191 (21.72%)
Faus‐Matoses et al. (2020)	Cross sectional, observational study.	Valencia	Clínic	251	Mean: 16 Range 1–77	Males: 157 (62.5%) Females: 94 (37.5%)	Andreasen	Deciduous Permanent Mixed	Avulsion Luxation Fracture Intrusion Concusion Extrusion Subluxation	Not available
Veloso Duran et al. (2022)	Cross sectional, observational study.	Barcelona	Clínic	1400	Groups: 3–5 6–8 9–11 12–14	Males: 733 (52.4%) Females: 667 (47.6%)	Not available	Deciduous Permanent Mixed	Not specified	63 (4.5%)

Abbreviation: TDI, traumatic dental injuries.

### 3.3 Assessment of Methodological Quality

**Figure 2:**
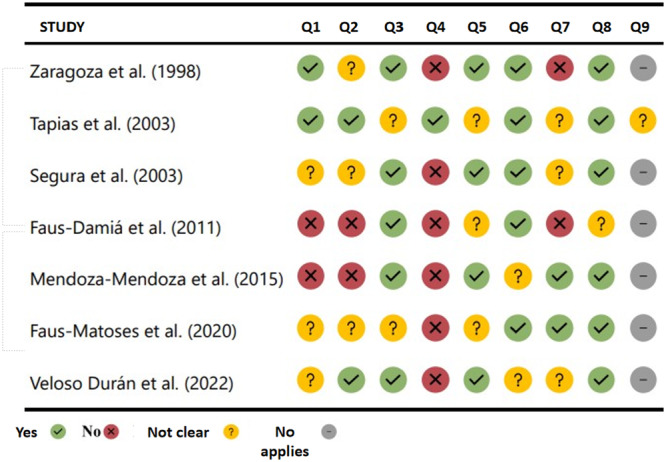
Summary of the assessment of the methodological quality of the included studies. *Note:* Q1: Was the sampling frame appropriate to address the target population; Q2: Was an adequate sample of study participants drawn; Q3: Was the sample size adequate; Q4: Were the study subjects and setting described in detail; Q5:Was the data analysis conducted with sufficient coverage of the identified sample; Q6: Were valid methods used for the identification of the condition under study; Q7: Was the condition measured in a standard and reliable way for all participants; Q8: Was an adequate statistical analysis performed; Q9: Was the response rate adequate? If not, was the low response rate adequately managed?

The assessment of the methodological quality of the included studies showed several inconsistencies in the applied methodology. There were major inconsistencies in the description of the sampling frame and in the characterization of the participants, which calls into question the representativeness of the reported results. In addition, most of the studies failed to adequately identify and control for possible biases, such as selection and classification biases, which affects the internal validity of the findings. On the other hand, most studies presented an adequate sample size and applied appropriate statistical analyses to estimate prevalence (Figure 2).

### 3.4 Prevalence of TDI

The overall estimated prevalence of dentoalveolar trauma in the Spanish population was 9.94% (95% CI: 5.98%–16.6%). The prediction interval of the prevalence was 1.42%–45.6%, indicating high heterogeneity between studies.

**Figure 3:**
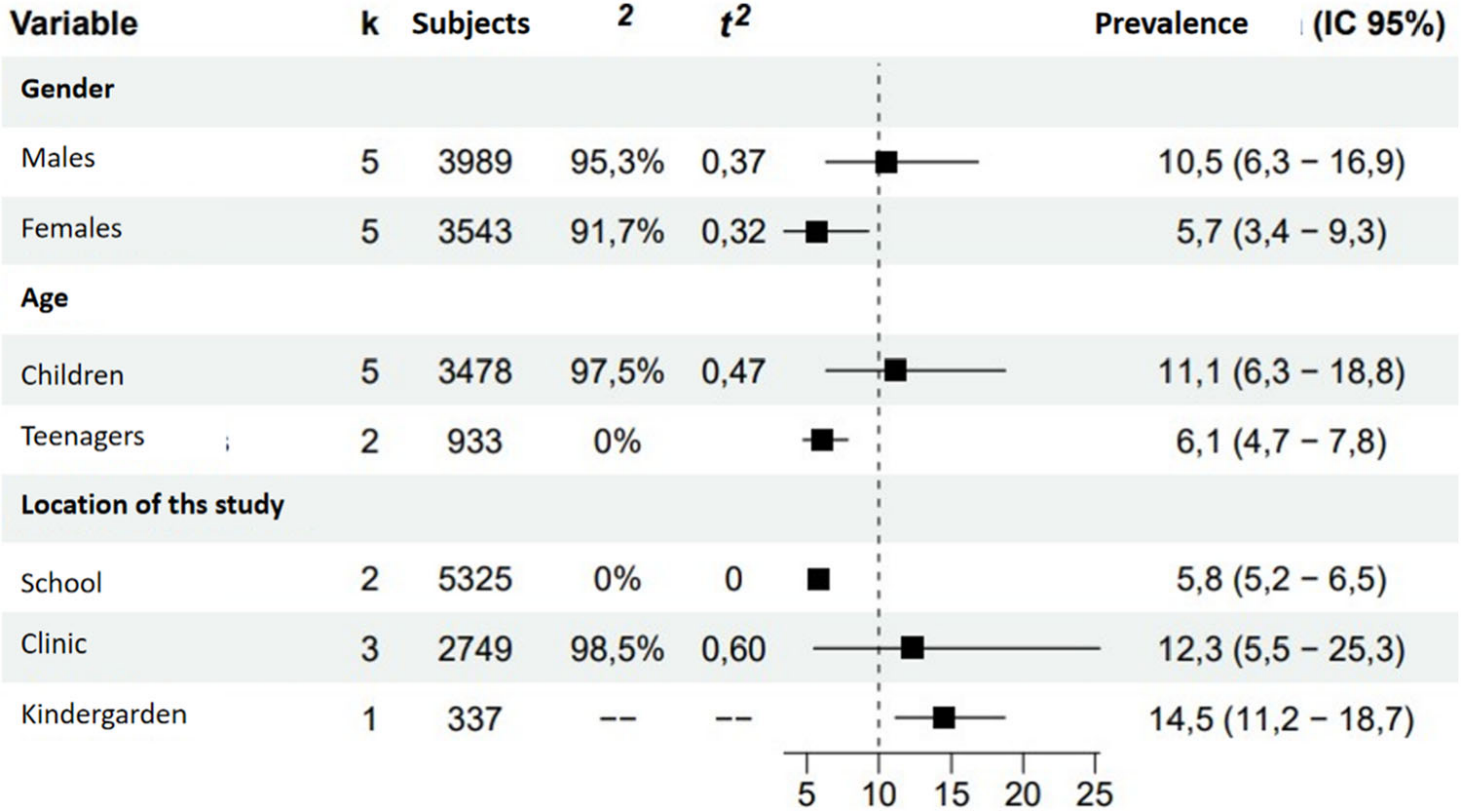
Prevalence of dentoalveolar trauma by subgroups. *Note:* k: number of studies. Prevalence is expressed as a percentage. CI, confidence interval.

The results of the subgroup analysis indicated that the prevalence of dentoalveolar trauma was higher in male participants (10.5% [6.3%–16.9%]) compared to female participants (5.7% [3.4%–9.3%]), as well as in children (11.1% [6.3%–18.9%]), and in children (11.1% [6.3%–18.6%]) compared to adolescents (6.1% [4.7%–7.8%]). It was not possible to estimate the prevalence of TDI in adults. Studies conducted in day‐care centers and dental clinics reported higher prevalences than those conducted in school settings. High heterogeneity was observed in most of the subgroups analyzed, particularly in studies conducted in clinical settings and in the children's group, suggesting considerable variability between the studies included in the review (Figure 3).

**Figure 4:**
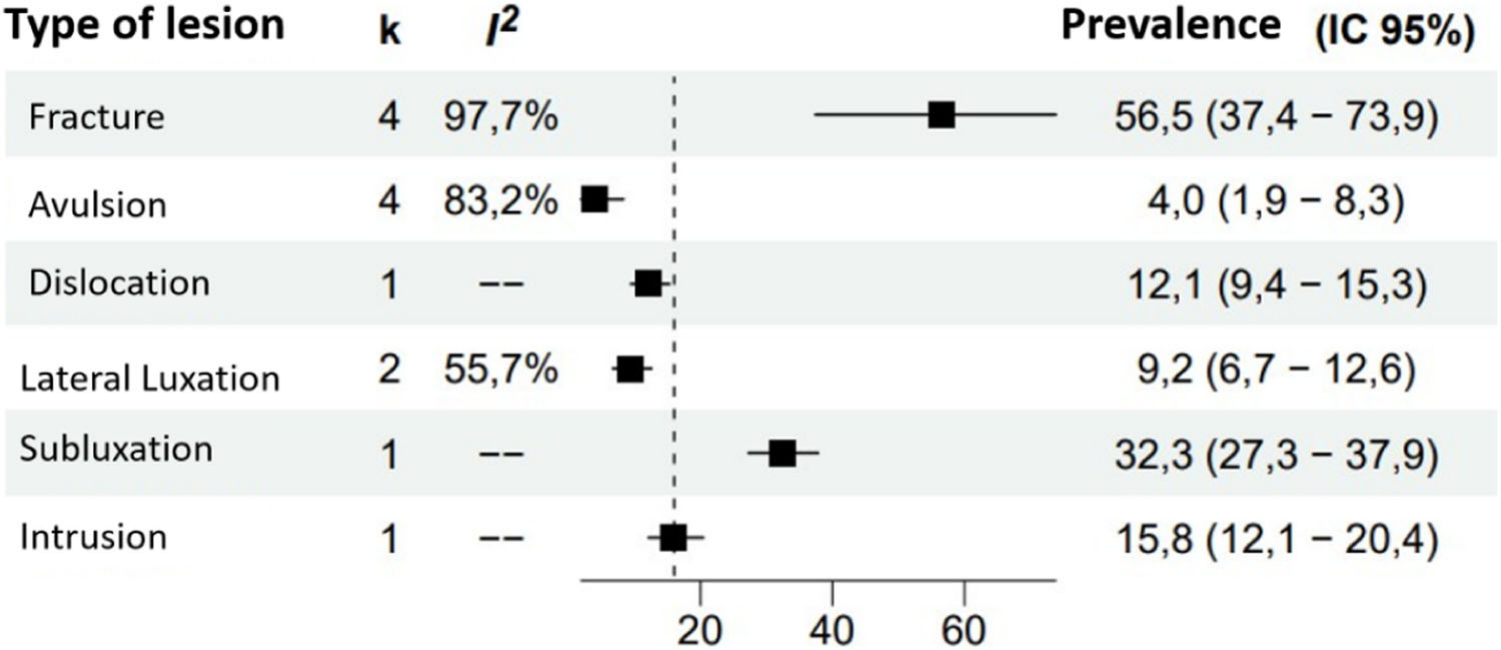
Prevalence of the different types of dentoalveolar trauma. *Note:* k: number of studies. Prevalence is expressed as a percentage. CI, confidence interval.

Fracture was the most frequent type of trauma, with a prevalence of 56.5% according to four studies, although with considerable heterogeneity. On the other hand, avulsion had a much lower prevalence of only 4.0%, also with high heterogeneity. The remaining types of trauma, such as dislocation, subluxation and intrusion, showed prevalences of 12.1%, 9.2%, 32.3%, and 15.8%, respectively, although these results are from one or two studies per type and show variability in the heterogeneity reported (Figure 4).

## 4 Discussion

The results of this systematic review and meta‐analysis provide a comprehensive overview of the prevalence of TDI in the Spanish population, highlighting variations by gender, age and study setting. The overall estimated prevalence of TDI was 9.94%, which is in line with previous studies (Petti, Glendor, et al. 2018) reporting a prevalence in Europe of between 9.8% to 18.9%, albeit with a high degree of heterogeneity among the included studies. This high degree of heterogeneity may be attributed to differences in data collection methods, inclusion criteria and classification of TDI, as well as the diversity of settings in which the studies were conducted.

The results indicated a higher prevalence of TDI in males (10.5%) compared to females (5.7%). This finding has also been reported by other authors. Petti, Glendor, et al. (2018). found that the prevalence in the European region in men was 1.48 (95% CI: 1.20–1.83) times higher than in women, a result that was consistent with other regions of the world. Another author (Aldrigui et al. 2014), in a systematic review and meta‐analysis conducted to estimate the prevalence of TDI in Southamerica, also found that men were more at risk than women for TDI (OR: 1.72 [95% CI: 1.57–1.89]). This finding could be explained by the greater involvement of males in risky physical activities and more aggressive behaviors.

The higher prevalence of TDI in children (11.1%) compared to adolescents (6.1%) can be explained by several factors associated with children's physical development and behavior. In younger children, the lack of full development of fine motor skills and coordination makes them more prone to falls and accidents, especially during the early stages of childhood. These accidents often occur during everyday or recreational activities, such as play at home or at school, which increases the risk of TDI. However, the evidence is contradictory. On the one hand, Aldrigui et al. (2014) argue that injuries are cumulative events, so that the prevalence of injuries is expected to be higher in older populations; while, on the other hand, other studies (Lembacher et al. 2022; Andreasen and Ravn 1972; Slayton and Palmer 2019) suggest that the increased vulnerability of young children to everyday accidents and the lack of adequate protective mechanisms, such as limited use of mouthguards or constant parental supervision, counteract this accumulation of risk. Thus, while it is true that adolescents may have accumulated increased exposure to risky situations over time, the nature of childhood activities, combined with physical and developmental factors, appears to predispose children to a higher frequency of TDI compared to adolescents.

Studies conducted in clinical and day care settings reported higher prevalences than those conducted in school settings, suggesting that the prevalence of TDI may be influenced by the setting in which the study was conducted, with possible biases towards higher detection in settings where monitoring of individuals' general health is systematic. This variability highlights the need for a standardized approach to the assessment and recording of TDI to facilitate comparability across studies and regions.

Dental fracture, as in several studies (Gopinath et al. 2008; Gojanur et al. 2015), emerged as the most frequent traumatic injury with 56.5% of TDIs. On the other hand, although avulsion was considerably less frequent, occurring in only 4.0% of cases, it remains a highly serious injury due to the difficulty of successfully reimplanting the avulsed tooth and the high failure rates of these procedures, especially if proper management is not performed in the critical time after trauma (Fouad et al. 2020).

This study has several clinical and public health implications. The findings underline the importance of implementing preventive and educational programmes, especially targeting at‐risk populations, such as young children and adolescents, and in settings where a higher prevalence of TDI is observed. Furthermore, the need to improve data collection and uniformity in the classification of TDI to provide more accurate and comparable estimates is highlighted.

The systematic review has several notable strengths. It is the first known study that attempts to estimate the prevalence of TDI in Spain, using a comprehensive search strategy that includes grey literature and alternative methods of reference identification. In addition, a data mining tool based on machine learning was used, which improved the efficiency of article selection (Li et al. 2023). The JBI tool for the evaluation of prevalence studies allowed for the comprehensive assessment of studies, analyzing key aspects such as representativeness of the sample, measurement precision and internal validity, ensuring that the assessment of the implications of the findings is done within an appropriate framework. In addition, adherence to PRISMA guidelines reinforces the transparency and reproducibility of the methodological process.

However, the study faces significant limitations, such as the high heterogeneity among the included studies, the lack of standardization in the classification of TDI and the study settings, as well as methodological shortcomings in the analyzed studies and study contexts, as well as methodological shortcomings in the studies analyzed. These limitations may affect the generalizability and accuracy of the results obtained, underlining the need to interpret them with caution and to improve methodological quality in future research.

Future studies seeking to estimate the prevalence of TDI should adopt a more standardized and methodologically rigorous approach to overcome the limitations identified in this review. First, the use of a uniform and internationally validated classification system for TDI, such as the WHO‐approved NAOD classification (Petti et al. 2022), is essential and would facilitate comparison of results across studies and regions. Furthermore, it is crucial that future studies use a clearly defined sampling frame that is representative of the target population, ensuring the inclusion of diverse regions and socioeconomic contexts to improve the generalizability of findings. It is also recommended that longitudinal study designs be implemented to assess temporal trends in TDI.

## 5 Conclusions

This systematic review provides an estimate of the prevalence of dental trauma in Spain, showing a high frequency of these events, especially in children and adolescents. Although this study provides valuable information, the methodological limitations identified in the existing literature underline the need for future research with more standardized and rigorous approaches, which will allow more precise and comparable estimates of the prevalence of dental trauma to be obtained.

## Author Contributions

All authors contributed equally to the publication of the article.

## Conflicts of Interest

The authors declare no conflicts of interest.

## Data Availability

The data that support the findings of this study are available from the corresponding author upon reasonable request.
